# Spirituality in the uncertainty of illness: the perspective of oncology patients

**DOI:** 10.1590/0034-7167-2022-0712

**Published:** 2023-10-09

**Authors:** Roberto Firpo de Almeida, Maria Cristina Soares Figueiredo Trezza, Isabel Comassetto, Lucas Kayzan Barbosa da Silva, Marcela Porangaba Lopes, Kleytonn Giann Silva de Santana, Francyane Adielle de Souza Praxedes

**Affiliations:** I Universidade Federal de Alagoas. Maceió, Alagoas, Brazil; II Centro Universitário CESMAC. Maceió, Alagoas, Brazil

**Keywords:** Spirituality, Nursing Theory, Neoplasia, Uncertainty, Patient., Espiritualidad, Teoría de Enfermería, Neoplasia, Incertidumbre, Paciente., Espiritualidade, Teoria de Enfermagem, Neoplasia, Incerteza, Paciente.

## Abstract

**Objective:**

To analyze spirituality in the process of illness uncertainty in cancer patients.

**Methods:**

This is a qualitative study, in which Merle Mishel’s Theory of Uncertainty of Disease was used as a theoretical framework; and as a methodological reference, the stages of Bardin’s Content Analysis. As a technique for obtaining information, a semi-structured interview was used.

**Results:**

Spirituality in the uncertainty of the disease varies from patient to patient and acts in a unique way. They presented readaptation attitudes in their reports. The presence of spirituality in their lives acted as the main force mechanism to deal with the uncertainty of the disease, and this moment was called by Mishel “probabilistic thinking”.

**Conclusion:**

Patients demonstrated readaptation attitudes in their reports, and spirituality acted as the main mechanism of strength to deal with uncertainty in the disease

## RESUMO

**Objetivo:**

Analisar a espiritualidade no processo da incerteza da doença no paciente oncológico.

**Métodos:**

Trata-se de uma pesquisa qualitativa, na qual foi utilizada como referencial teórico a Teoria da Incerteza da Doença, de Merle Mishel; e como referencial metodológico, as etapas da Análise de Conteúdo de Bardin. Como técnica de obtenção das informações, foi usada a entrevista semiestruturada.

**Resultados:**

A espiritualidade na incerteza da doença varia de paciente para paciente e atua de forma singular. Eles apresentaram atitudes de readaptação nos seus relatos. A presença da espiritualidade em suas vidas agiu como o principal mecanismo de força para lidar com a incerteza da doença, sendo, esse momento, chamado por Mishel de “pensamento probabilístico”.

**Conclusão:**

Os pacientes demonstraram atitudes de readaptação nos seus relatos, e a espiritualidade atuou como o principal mecanismo de força para lidar com a incerteza na doença.

## RESUMEN

**Objetivo:**

Analizar espiritualidad en el proceso de la incertidumbre de la enfermedad en el paciente oncológico.

**Métodos:**

Investigación cualitativa, en la cual fue utilizada como referencial teórico la Teoría de la Incertidumbre Frente a la Enfermedad, de Merle Mishel; y como referencial metodológico, las etapas del Análisis de Contenido de Bardin. Como técnica de obtención de las informaciones, fue usada la entrevista semiestructurada.

**Resultados:**

La espiritualidad en la incertidumbre de la enfermedad varia de paciente para paciente y actúa de manera singular. Ellos presentaron actitudes de readaptación en sus relatos. La presencia de la espiritualidad en sus vidas actuó como el principal mecanismo de fuerza para lidiar con la incertidumbre de la enfermedad, siendo, eso momento, llamado por Mishel de “pensamiento probabilístico”.

**Conclusión:**

Los pacientes demostraron actitudes de readaptación en sus relatos, y la espiritualidad actuó como el principal mecanismo de fuerza para lidiar con la incertidumbre en la enfermedad.

## INTRODUCTION

The spiritual dimension is little appreciated in health care, in nursing in particular. It becomes noticeable the need to consider the care of this dimension to constantly deal with the uncertainties of the disease; however, assistance, in the conventional service, is provided primarily with an emphasis on the biological dimension.

Historically, Plato was the first philosopher to speak of a spiritual intuition, an understanding that was also followed by Plotinus, Saint Augustine, Descartes (with his “I think, therefore I am”), among other thinkers^(1)^. Defining spirituality and its relationships, such as religion and religiosity, is a complicated task, because, as Delfino^(2)^ points out: “there is no definitive and consensual concept of spirituality in the literature. Perhaps because it is a complex and deeply human notion.” With the theoretical diversity and considering the approximation with the holistic vision of health, the conceptions about spirituality, whose focus was on the “meaning of life”, bring as an example the definitions of Siqueira et al.^(3)^:

One of the dimensions of the human experience. It is expressed by the inner search of human beings and by the constructed meaning, through their beliefs, values, and principles, which can rescue the meaning of life and, thus, enable interrelationships with the divine, with nature and with oneself.

These themes have brought people closer to God and faith, making them strong allies in the face of diseases, especially serious ones, such as cancer^(4)^.

**Figure 1:**
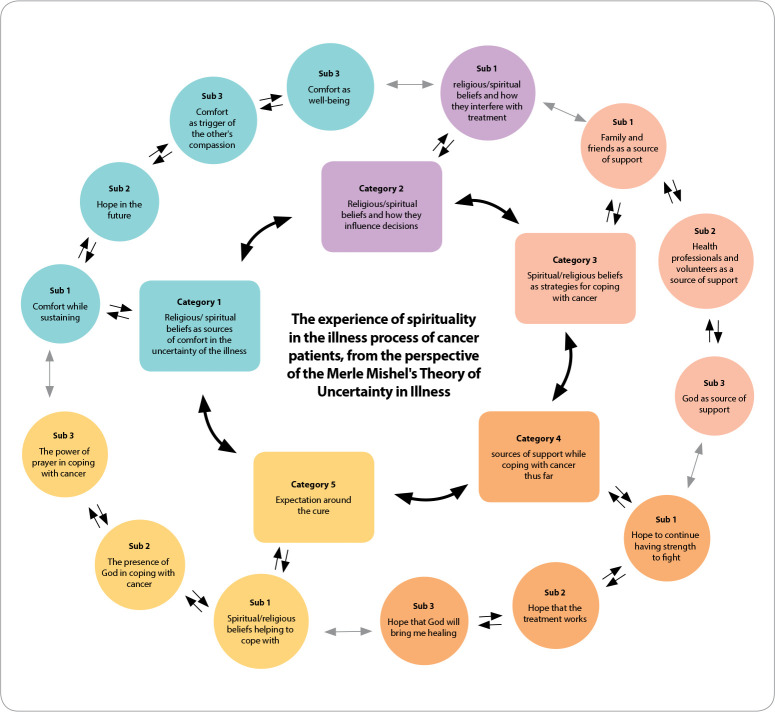
Flowchart of categories and subcategories arising from content analysis according to Bardin

When considering religion as an organized system of beliefs, practices, rituals and symbols created to facilitate approximation with the sacred or transcendent (God, greater power, ultimate reality)^(5)^, it can be said that it is about institutionalized spirituality. Religiosity is the religious practice itself, such as the act of going to church, to services, and practicing the rituals that are part of a certain religion^(6)^. In their studies, Inoue and Vercina^(7)^ as well as Figueiredo^(8)^ reveal that patients would like their religiosity/spirituality (R/S) to be addressed in clinical practice and clinicians recognize the importance of this approach, as few professionals effectively question their patients about this aspect of their lives^(6)^. Sousa^(9)^ states that 59% of British medical schools and 90% of American ones have courses or contents on spirituality and health, while, in Brazilian medical schools, there is a gap between students’ attitudes and expectations regarding the inclusion of religiosity/spirituality in the their training and clinical practice^(9)^.

The oncological disease involves aspects such as physical pain, submission to complex treatments and the uncertainty associated with the prognosis of life or death. When faced with the diagnosis of cancer, the individual can trigger a psychic, physical, social, emotional and spiritual imbalance, which causes strong impacts on his life^(10)^. Filho and Khoury^(11)^ say that it is necessary to mobilize psychosocial and spiritual resources to deal with the suffering and adverse effects of cancer; this feature is known as “coping”.

Coropes^(12)^ points out that spirituality, as a resource for coping with cancer, is a step in which it is possible to make human beings’ vulnerability and their own perception of finitude more visible, which may be related to the need for spiritual support. In a survey carried out with health professionals in a hospital institution, a reference in palliative care, 94.8% of the evaluated professionals believe that the theme “Health and Spirituality” should be part of the regular curricula of health education.

According to the National Cancer Institute^(13)^, for Brazil, the estimate for each year of the three-year period 2023-2025 is that there will be 704,000 new cases of cancer (450,000, excluding cases of non-melanoma skin cancer)^(14)^.

From the perspective of a new model of assistance that could attribute importance to the establishment of spiritual care by nursing professionals, research is needed that embraces this challenge in order to consolidate health promotion as a pillar of this care. Based on what has been exposed, this research presents the following guiding question: “How does spirituality show itself in the process of illness of cancer patients according to the Nursing Theory of Uncertainty in Illness, by Merle Mishel?” The importance of this study resides in the presentation of a model of care for cancer patients that applies a nursing theory, specifically Merle H. Mishel’s theory, of medium range; it addresses the uncertainties, anxieties and doubts of the patient himself, his caregivers and family members in relation to a current health problem, in its acute or chronic phase. The theory reports that high levels of uncertainty are associated with reduced skills such as processing new information, understanding results, and adapting to the diagnosis of the disease.

## OBJECTIVE

To analyze spirituality in the process of illness uncertainty in cancer patients.

## METHODS

### Ethical aspects

The research met the requirements of the Standards for Reporting Qualitative Research (SRQR) instrument recommended by the EQUATOR network for qualitative studies. The research project, originated for a master’s thesis, was approved by the Research Ethics Committee, following Resolutions of the National Health Council nº 466 (of December 12, 2012) and nº 510 (of April 7, 2016), with participants being guaranteed compliance with the precepts of autonomy, beneficence, non-maleficence, and justice. Data collection took place after the participants’ consent by signing both copies of the Free and Informed Consent Form (FICF). Anonymity was guaranteed by replacing names with the letter P (participant) followed by a cardinal number, according to the sequence of interviews. The confidentiality of the testimonies is assured, under the custody of the main researcher for five years.

### Study type

This is a qualitative study with a descriptive approach, since it works with the universe of meanings, motives, beliefs, values and attitudes, which cannot be reduced to the operationalization of variables - in this specific case, with a comprehensive view of human life^(15)^.

### Study scenario and sample

Information gathering began in December 2020, after approval of the project by the Research Ethics Committee and was completed in February 2021. The research participants were 11 people with oncological disease, who were undergoing chemotherapy at the Centro de Alta Complexidade in Oncology (CACON), at the University Hospital Professor Alberto Antunes (HUPAA), Maceió, state of Alagoas (AL). The interview was face-to-face, with an average time of 40 minutes per participant. Inclusion criteria were defined as: oncology patients, on an outpatient basis, over 18 years old, who agreed to participate in the research. Those who were unable to communicate and with a very compromised clinical picture were excluded.

In search of similar studies that used the Theory of Uncertainty in Illness in cancer patients, with a focus on spirituality, only one research was found, namely, by Perdomo et al., with the title “Uncertainty before the diagnosis of cancer”, from the year 2018. It was carried out with six participants, which initially determined that the present study would have at least the same value.

### Production of information

The approach to the participants of this study, that is, cancer patients seen at the Center for High Complexity in Oncology (CACON), of the University Hospital Professor Alberto Antunes (HUPAA), on an outpatient basis, was spontaneous, with clarification on the purpose of the study and guarantee of anonymity; all were instructed about the possibility of interrupting the interview if they wanted to, without inducement or embarrassment. The information was produced using the interview technique, a collection method that allows access to descriptive data in the participant’s own language and allows the researcher to interactively develop an idea about how individuals interpret aspects of the world^(15)^.

Each interview was carried out in a single moment with each participant, following three steps, all completed by the author according to the responses collected: (1) collection of sociodemographic data, through a questionnaire prepared by the author; (2) obtaining a spiritual history, using the instrument already validated in Brazil, CSI-MEMO, consisting of four open questions; (3) the Disease Uncertainty Scale (Adult), based on the assumptions of Merle Mishel’s theory, with 30 questions containing answers classified by a Likert scale. An iPhone-type cell phone was used as an instrument for capturing information, which enabled full transcription and better analysis. The open, in-depth or semi-structured interview method was adopted^(16)^, with guiding questions that give the interviewer the freedom to develop situations and broadly explore the desired question.

### Data analysis

Bardin’s Content Analysis^(17)^ was used, which consists of a set of communication techniques aimed at obtaining, through systematic procedures and objective description of the content of messages, indicators (quantitative or not) that allow the inference of relative knowledge to the conditions of production/reception of these messages^(17)^. Bardin^(17)^ indicates that the use of content analysis foresees three fundamental phases according to the scheme shown in Figure 6: pre-analysis, material exploration and treatment of results (inference and interpretation). In addition, to discuss the analysis, Theory of Uncertainty in Illness was used, as a theoretical perspective capable of relating “the uncertainties experienced by patients in the face of the diagnosis of cancer and the role of nurses in caring for oncopatients” with “the concepts of patient-centered care”, as well as Mishel’s elements of “danger” and “opportunity”.

## RESULTS

Based on the principles of individuality and uniqueness of each participant, for a better understanding of the context of spiritual care, Chart 1 presents a summary of the sociodemographic characteristics of the study participants and their score.

**Chart 1:** Sociodemographic and personal factors of the research participants and total score obtained by measuring uncertainty in the disease

Patient	Sex	Age	Scholarity	Race/Color	Type of cancer	State /City	Religion	Total score
**P1**	M	63 years	Incomplete fundamental	Brown	Prostate cancer	Marechal Deodoro/AL	Evangelical	97
**P2**	F	56 years	Complete high school school	Black	Breast and colorectal cancer	Atalaia/AL	Spiritualist	104
**P3**	F	53 years	Incomplete fundamental	Brown	Breast cancer	Murici/AL	Evangelical	95
**P4**	F	27 years	Fundamental complete	Brown	Breast cancer	Propriá/SE	Evangelical	89
**P5**	F	44 years	Complete high school school	Brown	Stomach cancer	Maceió/AL	Catholic	91
**P6**	F	75 years	Illiterate	Brown	Breast cancer	Aliança/PE	Catholic	110
**P7**	F	47 years	Complete high school school	Brown	Pancreatic and liver cancer	Viçosa/AL	Catholic	93
**P8**	F	34 years	Complete high school school	Brown	Breast cancer	Maceió/AL	Evangelical	83
**P9**	F	46 years	Complete high school school	White	Lung cancer	São José da Laje/AL	Evangelical	91
**P10**	F	53 years	Postgraduate	Yellow	Acute myeloid leukemia	Água Preta/PE	Catholic	118
**P11**	F	69 years	Complete high school school	Brown	rectal cancer	Porto Calvo/AL	Evangelical	74

**Chart 2:** Summary of the spiritual anamnesis of cancer patients according to the CSI-MEMO

Questions	Summary of responses from the 11 survey participants
1) Do your religious/spiritual beliefs bring you comfort or are they sources of stress?	All reported religiosity/spirituality as a source of comfort. Most linked comfort to a word that involves God, a word that brings comfort to the soul, peace to the heart, reducing their sadness and uncertainty. Comfort revolves around faith in God, it is linked to some religion.
2) Do you have any spiritual/religious beliefs that may influence your medical decisions?	Three patients reported that religiosity/spirituality can influence medical decisions, associating belief in God, in their religion, with this decision. The others reported that it does not influence, saying that they are different things: God gave the gift to doctors; it is necessary to follow what they recommend; do not stop following the treatment so that God can help with the cure.
3) Are you a member of any spiritual or religious communities? Does she help you in any way?	Six patients reported being evangelical; four, Catholic; and one, spiritualist. Most reports show that they found comfort and help in prayer, in God, in Our Lady, in churches, family, friends, neighbors, health professionals, intersections and praises.
4) Do you have another spiritual need you’d like to talk to someone about?	Only one participant reported having a spiritual need that he would like to talk to someone about: he would like to know and listen more to the word of God. The others reported being satisfied with their spiritual needs; all linking their spirituality to religion and the presence of God.

Although there was no intention to screen the participants based on criteria related to the type of cancer, stage, sex, race/color, religion, etc., it is appropriate to point out the predominance of women in the research, since there was only one man. Among the participants, the age group from 40 to 75 years old stands out, and breast cancer as prevalent among women; regarding education, most had completed high school. Six participants underwent some type of surgery, all underwent or are undergoing chemotherapy and only one underwent radiotherapy. One participant, as a result of the surgery, used a colostomy bag.

The research participants were studied with the application of Mishel’s Uncertainty in Illness Scale^(18)^. In addition, the degree of uncertainty was measured using the adapted instrument, built based on this original scale by Mishel^(18)^. According to Mishel^(19)^, the score of each patient can vary between 30 and 150, whose higher value represents a greater degree of uncertainty in the illness: much or total uncertainty about their clinical evaluation. For the participants, the score varied between 74 (P11) and 118 (P10), therefore, all 11 presented an intermediate degree of uncertainty, as they approached the average value of 90.

CSI-MEMO is an acronym for the English words Comfort, Stress, Influence, Member of religious community, Other spiritual needs.)

Data analysis was developed based on the reading of the interviews referring to the participant’s characterization instruments and the obtaining of the patients’ spiritual history, in which the CSI-MEMO instrument and the interviewees’ speeches were used. Five major categories emerged from the testimonies of the participants; all with at least one subcategory.

**Category 1 - Religious/spiritual beliefs (R/S) as sources of comfort in the uncertainty of the illness:** person’s conviction to consider their religious and spiritual beliefs as a source of comfort, hope for the future, triggering compassion for others and well-being.

**Subcategory 1.1 - Hope for the future**: well-being by understanding that there is a sense of overcoming, which would give hope in the existence of a future after the disease, unveiled through faith and confidence to overcome obstacles, as can be seen in the following statements:

*Comfort because it makes me understand that what I’m going through has a meaning and that I’ll be able to overcome it* […]. (P5)

*Comfort because it connects me to the ultimate source of healing, that is, divine power.* (P7)

*Comfort is faith and hope in the future.* (P8)

**Subcategory 1.2 - Comfort as support and triggering compassion:** R/E providing comfort, strength, and incessant support during the illness process, especially when it is difficult to continue; comfort when he feels others intercede for him. Awakens compassion among people, resulting in intercession for the sick.

[…] *they are a source of comfort, you know, my religion is the one that I didn’t even say, right, evangelical. That’s where we get more attached, right? My faith is very big, and that’s what I’m feeding on, right? I believe even more.* (P3)

[…] *I will not get attached to a man, I get attached to God, he is the one who strengthens me, he is the one who gives me strength every day, and without him it is really difficult for us to continue* […]. (P9)

*Comfort because it leads others to intercede for me to make me understand God’s will for my life*. (P6)

**Subcategory 1.3 - Comfort as well-being**: translates spirituality/religion in the sense that its stimuli trigger joy, peace, tranquility and an indescribable lightness of the soul.

*Comfort is feeling good first. Because we have our choices, what is best for us, right?* […]. (P1)

*Comfort because the stimuli of my religion give me improvement, peace, and indescribable joy.* (P11)

**Category 2 - Religious/spiritual beliefs and how they influence decisions**: consists of people considering that their decisions are directly linked to their religious and spiritual beliefs, bringing consequences to their lives.

**Subcategory 2.1 - Religious/spiritual beliefs and how they interfere with treatment**: the belief that spirituality/religion, represented by God, will always interfere with treatment, due to the fact that physicians received from God the gift of treating in order to heal.

[...] *I have to follow what the doctors tell me, because I know that God gave them the gift, for them to do the treatment, for them to help me* [...]. (P9)

[...] *I will not stop doing the treatment because God will heal me* [...] *I have to do what the doctors say correctly, so that God will help me even more.* (P3)

**Category 3 - Spiritual/religious beliefs as strategies for coping with cancer**: spirituality itself - represented in faith in God, in the power of prayer, emanating a “strength” - can help to face the treatment, easing the pain and reducing their uncertainties in the face of cancer.

[…] *whenever I’m in pain, I always ask a lot of God, Our Lady. Sometimes they take away the pain without me taking the medicine, and that comforts me a lot, a lot, a lot, that God is the doctor of doctors.* (P8)

[…] *Yes, he helps, he visits me, he prays, he campaigns for, not financially, a spiritual aid campaign, of course, but that for me is a help that I think is greater than anything else.* (P10)

**Subcategory 3.1 - The presence of God in coping with cancer:** the presence of God healing, responding to the request for healing, if it is in His plans, accepting His will and power, having faith in better days, the certainty of even a miracle.

[…] *God is the one who comforts the hearts of all of us, because without God we are nothing, so every day I ask God to give me my healing. And that’s how I see it, slowly, but it’s good. It’s all in God’s plans, it’s all as God wants it and God sends it and we’re accepting it. We have to accept it, because God is the doctor of doctors, right?* (P8)

[…] *I believe in God, I am a believer and I am sure that I will be completely fine. I’m going to preach in church, I’m going to worship in thanksgiving, amen, you can be sure, I’m going to do that.* (P10)

[…] *right at the beginning, which is the most you get knocked down, it’s that red, the red chemo, I stayed in bed myself* [...], *that’s when I started to cry, but I got up, and not to mention the hand that I felt heavy in on my chest, about three times, I tried to get up, but I couldn’t, with a weight on my chest: it was God healing me. Okay, from that day on, I will never lay down again, never again.* (P3)

**Subcategory 3.2 - The power of prayer in coping with cancer:** prayer-from churches, friends, and family-and faith in God will restore health.

[…] *there are several churches praying for me, churches outside. There’s my family, friends, neighbors. I have friends who pray, they pray in the morning with their knees on the ground for me, friends, family, many people, that’s why I’m sure, my faith in God, and I feel that he will restore my health, this certainty I have, thank God.* (P11)

[…] *She helps me with words, it’s already a food, right? I think it helps me a lot. Words and prayers alone are enough.* (P3)

**Category 4 - Sources of support while coping with cancer so far:** support from family, friends, health professionals and especially God in the journey since the diagnosis.

**Subcategory 4.1 - Family and friends as a source of support or not:** having found, or not, family members and close friends as the main source of support throughout the illness process:

[…] *my comfort was my family: my children, my husband, my sisters, who were always with me.* (P9)

*I had help from everyone, family, and friends.* (P4)

*My daughter came for a few days* […] *psychologically, emotionally, she is too fragile. She was already getting nervous. My youngest son, who is 17 years old, is another anxious one. So, I prefer people by my side to make me feel safe, to make me nervous, I prefer no one to come* […]. (P2)

**Subcategory 4.2 - Health professionals and volunteers as a source of support:** positive feeling of contact, during illness, with health professionals and volunteers as sources of support.

[…] *my comfort was the professionals, you know, in the health area, who also helped me a lot; and the pink house that supported me and supports me until today*. (P9)

**Subcategory 4.3 - God as a source of support:** understand that spirituality and religiosity are linked to God, as the one and only one able to welcome, promote comfort and help in whatever is necessary.

*What has helped me the most so far is, first of all, God, my family, friends and the people inside, who are very welcoming.* (P2)

*God is my only source of support.* (P11)

*Help in everything, first of all God, and her (wife) by my side. That helps and everything* […]. (P1)

**Category 5 - Expectation around the cure:** hope to continue having strength to fight, that the treatment works and God brings the cure.

**Subcategory 5.1 - Hope to continue having the strength to fight and that the treatment works:** Belief that everything will go well, that changes in life and way of thinking are expected, as well as living with the uncertainty of the morning, for which one must be prepared.

[…] *I hope to live longer, to have many changes in my life, things that I used to think about in one way and now I think about it in another way. So, like that, it opened up some parts of my vision that I didn’t have before and today I have.* (P8)

Faced with the reports, the participants cling to the hope of healing, trusting in the arrival of better days, and this hope gives strength to continue.

*I hope that I get better, that I continue the treatment, I don’t know for how long, but I hope for the cure that I ask God for every day. Because it’s already the second time, I ask that God heal me, that I keep treating myself, because it’s for an indefinite period and to achieve the cure.* (P2)

*I hope that, soon, I will be able to have the transplant and I will continue to do the treatment as it should be after the transplant, because I have an indication for the transplant. Then, we already did some tests in the family there, and it didn’t work, but now I hope to get the donor and do it and then I can transplant, continue here as it has to be, and get well, of course.* (P10)

**Subcategory 5.2 - Hope that God will bring me healing:** it means believing in God as the main person responsible for the patient’s cure, since He knows the purpose and meaning of life, He is the hope, He is the cure:

*Ah, I hope to get well, because I believe. That God has purpose in my life and that’s why I will be well in Jesus’s name.* (P5)

*I hope you recover soon, right? Because it’s not easy to spend a year with this suffering, because it’s a lot of suffering, see? Only God. But God is faithful and true, God is healing.* (P7)

## DISCUSSION

In this study, there was a predominance of female interviewees (90%), ranging in age from 40 to 75 years. All responded that they had religious/spiritual beliefs, the majority being evangelical. As for education, most had completed high school, and only one participant reported being illiterate. Mishel^(19)^, in his Theory of Uncertainty in Illness, speculates that the level of formal education provides a cognitive resource for understanding disease-related information.

The author^(20)^ argues that when an event produces uncertainty in patients, especially patients with a chronic disease such as cancer, ambiguity emerges, so that they do not have sufficient skills to understand the real meaning of the disease and its implications. Alves^(21)^ states that, for cancer patients to better accept the condition they are in and to deal with a new situation, it is necessary to obtain as much information as possible.

As for the aspects related to the category “Religious/spiritual beliefs as a source of comfort in the uncertainty of the illness”, spirituality/religiosity is a source of comfort as support, hope for the future, triggering compassion and well-being. In his study, Arrieira^(22)^ states that faith brings comfort in coping with difficult situations experienced by cancer patients and their families; these people, faced with insecurity and sadness, find in their beliefs support for coping and answers to questions about living. Similarly, in the study by Barros and Morais^(23)^, it was seen that faith and spirituality provide better internal control in the face of fearful and uncertain situations.

Despite the different manifestations of religiosity/spirituality as a form of comfort, there was unanimous recognition of this comfort and help received through religious/spiritual beliefs. Oliveira^(24)^ links the improvement in quality of life and comfort to spirituality, indicating that a multidisciplinary intervention that includes a spiritual component led by a chaplain has a significant impact on the quality of life of cancer patients. Vidal et al.^(25)^ discuss in their study how spirituality is important because patients find meaning in life in the midst of uncertainty, reducing the traumas related to the disease.

As for the aspects related to the category “Religious/spiritual beliefs and the way they influence decisions”, the participants of this research gave the meaning that the spirituality/religiosity represented by God will always bring positive interference in the treatment, because the doctors received from Him the gift of treating for healing. Actions in favor of managing uncertainty are highly effective in managing this feeling. Júnior and Teixeira^(26)^ say that understanding spirituality is to differentiate it from the strictly religious aspect and implies respect for different beliefs and cultures, as this also becomes a tool for humanized care, in order to provide the professional with greater possibilities of assistance and healing. Liberato and Macieira^(27)^ state that spirituality can influence the way the patient faces the process of falling ill and its repercussions, as well as the way he attributes meanings to the illness and the complications experienced in the course of treatment.

Nursing has an important role in spiritual care, as it must help patients to reconnect with something that gives them support and strength during treatment. Regarding the category “Religious/spiritual beliefs as strategies for coping with cancer”, the research participants considered that spiritual/religious beliefs help to face the oncological disease and that the presence of God and the power of prayer make all the difference during the entire disease process.

Some studies have been carried out in nursing in the context of chronic diseases, revealing a relationship between spiritual and religious variables. A considerable relationship was found between these variables and feelings of well-being, quality of life, way of dealing with the disease, meaning and hope and social support^(28)^, since spirituality provides a fighting force for individuals with cancer face the whole process of uncertainties, illness and treatment^(29)^.

For Mishel^(30)^, uncertainty can be assessed as a danger or as an opportunity; and, depending on how it is analyzed, different strategies will be used. Health professionals can help patients find coping strategies in order to improve the conduct of treatment and the clinical situation of the patients’ illness^(19)^.

For Benites et al.^(31)^, hope attributes meaning to existence; hope is the search for the meaning of life, even in the face of death. Values such as love, joy, forgiveness, hope and compassion are one of the fuels of spirituality and reside in the sense of humanity, friendship and family, and can be contemplated in any human social action. Thus, spiritual well-being can significantly help in coping with the anguish related to the disease, as well as in promoting the mental health of the family members of the person with cancer^(32)^.

Suffering and spiritual pain are experienced by cancer patients, and spirituality is a solitary and intrinsic phenomenon, which encompasses the needs of human beings and solidifies an already established belief. In his study, Oliveira^(24)^ evaluated that prayer induces a feeling of relief from our tensions, because, when one prays, the mind focuses on a specific purpose, freeing itself from negative thoughts and day-to-day concerns. Arrieira^(22)^ says that religions offer solutions to the dilemma of death, generally associated with the presence of God; in this context, religious beliefs and practices meet the person’s need to have an expectation for the future^(22)^. In studies that point to the relationship between spirituality, maintenance of hope and attribution of meanings to the disease and to life, it is shown that spirituality provides a sense of control and alleviates the suffering experienced^(27-30,32)^.

In a survey carried out by Figueiredo et al.^(33)^, it was demonstrated that participants with a chronic illness have their family members as a source of comfort (social support), who are the main strength to deal with a chronic illness and encourage them to the treatment^(33)^. For Mishel and Clayton^(34)^, support from family, friends and people with similar experiences reduces uncertainty. Data from the study by Balboni^(35)^ and Saad & Medeiros^(36)^ found similarities with the concept of the subcategory “God as a source of support”. Spirituality, in the face of adverse situations, can represent a source of comfort, well-being, security, meaning and strength.

Regarding the category “Expectation around the cure”, spirituality is seen as a strength to continue fighting; moreover, regarding this category, the role of faith and its contribution to these people to overcome the difficult moments throughout the cancer diagnosis. Faith is associated with the feeling of hope, well-being, and security that God will provide the cure at some point and represents a way of coping.

According to Mishel^(30)^, one of the factors that influence the formation of a new perspective on life is health professionals. Their information promotes a probabilistic view of the world, encouraging the development of a new view of order in the patient. Nurses’ activities in chronic diseases normally work to promote probabilistic thinking, so it is necessary to highlight the importance of health professionals not ignoring the spiritual aspect of care. Such care, when promoted by professionals, is perceived by the physiognomy when welcoming, by the attention, by the love they express in their actions - which is manifested in the participants’ speeches. These attitudes help in the treatment.

### Study limitations

The limitations refer to: the gender of the participants, since the female gender made up the majority (it is noted that only one man was part of the sample); the study setting, as data were collected only at the chemotherapy outpatient clinic, due to the pandemic situation and impediment to visits to wards and home; to the time of collection, with a delay of months, which made it difficult to deepen and diversify the information beyond participants who had not always linked their spirituality to an institutionalized religion. Within the scope of the Theory of Uncertainty of the illness, the limitations emerged from questions that were not explained and that arose with the development of the work, but there was no reliable data; or, due to lack of data, such questions could not be answered.

### Contributions to the area of nursing, health, or public policy

As a product of this study, health professionals, proponents of public policies and especially nurses are offered the knowledge that the Nursing Theory of Uncertainty in Illness is capable of subsidizing information or even inspiring new research in the search for improved spiritual care to chronic patients - especially those affected by cancer, in whom a high prevalence of uncertainty was found - so that this theoretical foundation can be increasingly applied to the reality of Brazilian nursing.

## CONCLUSIONS

Spirituality in the uncertainty of the illness varies from participant to participant and acts in a unique way in each one. The patient is able to live and deal with their uncertainties in the perspective that it will result in the cure or calming down of their suffering. Spirituality is shown to be important in the cancer patient’s illness process; it is noteworthy that the patients showed readaptation attitudes in their reports, and the spirituality in their lives acted as the main mechanism of strength to deal with the uncertainty of the illness. This moment is called by Mishel “probabilistic thinking”: the patient manages to live and deal with his uncertainties in the hope of receiving and having as a result the cure or the calming of his suffering.

Merle Mishel’s theory proved to be clear, simple, precise, and general, attesting to its ability to be applied to patients affected by a chronic disease - in this study, cancer. Thus, it made it possible to identify the impacts of spirituality on the uncertainties experienced by the participants. Reading the content of the interviewees’ statements in the light of this theory also provided an understanding of the antecedents of these patients’ uncertainty, ways of coping with the disease, ways of adapting and the participants’ assessments of their current conditions. It is also emphasized the importance of family, friends, and health professionals in facing uncertainty or in adapting to it, acting as providers of structures (social support, credible authorities), according to the Theory of Uncertainty in Illness.
